# Charge collection efficiency, underlying recombination mechanisms, and the role of electrode distance of vented ionization chambers under ultra-high dose-per-pulse conditions

**DOI:** 10.1016/j.ejmp.2022.10.021

**Published:** 2022-12

**Authors:** Rafael Kranzer, Andreas Schüller, Faustino Gómez Rodríguez, Jan Weidner, Jose Paz-Martín, Hui Khee Looe, Björn Poppe

**Affiliations:** a PTW-Freiburg (R&D), Freiburg 79115, Germany; b University Clinic for Medical Radiation Physics, Medical Campus Pius Hospital, Carl von Ossietzky University Oldenburg, 26121, Germany; c Departamento de Fisica de Particulas, Universidad de Santiago, Santiago de Compostela, Spain; d Laboratorio de Radiofisica, Universidad de Santiago, Santiago de Compostela, Spain; e Physikalisch-Technische Bundesanstalt, Braunschweig 38116, Germany

**Keywords:** Dosimetry, FLASH radiotherapy, Ultra-high dose-per-pulse, Ionization chamber

## Abstract

**Purpose:**

Investigating and understanding of the underlying mechanisms affecting the charge collection efficiency (CCE) of vented ionization chambers under ultra-high dose rate pulsed electron radiation. This is an important step towards real-time dosimetry with ionization chambers in FLASH radiotherapy.

**Methods:**

Parallel-plate ionization chambers (PPIC) with three different electrode distances were build and investigated with electron beams with ultra-high dose-per-pulse (DPP) up to 5.4 Gy. The measurements were compared with simulations. The experimental determination of the CCE was done by comparison against the reference dose based on alanine dosimetry. The numerical solution of a system of partial differential equations taking into account charge creations by the radiation, their transport and reaction in an applied electric field was used for the simulations of the CCE and the underlying effects.

**Results:**

A good agreement between the experimental results and the simulated CCE could be achieved. The recombination losses found under ultra-high DPP could be attributed to a temporal and spatial charge carrier imbalance and the associated electric field distortion. With ultra-thin electrode distances down to 0.25 mm and a suitable chamber voltage, a CCE greater than 99 % could be achieved under the ultra-high DPP conditions investigated.

**Conclusions:**

Well-guarded ultra-thin PPIC are suited for real-time dosimetry under ultra-high DPP conditions. This allows dosimetry also for FLASH RT according to common codes of practice, traceable to primary standards. The numerical approach used allows the determination of appropriate correction factors beyond the DPP ranges where established theories are applicable to account for remaining recombination losses.

## Highlights

• Deeper understanding of the charge collection efficiency of ionization chambers.
• Simulation of the charge collection efficiency including charge multiplication.
• Application of ionization chambers under ultra-high dose rate electron beams.
• Promising tool for real-time dosimetry in FLASH radiotherapy.

## Introduction

Currently, ultra-high dose rates (UHDR) are used to study the so-called FLASH effect. It was found that irradiation with UHDR greater than 40 Gy/s can lead to sparing of healthy tissue while maintaining tumor control. This sparing effect has been discussed and reviewed recently in the literature [1], [2], [3], [4], [5]. Most studies of this biological effect have been performed with dedicated electron linear accelerators or modified medical accelerators with pulsed beams [6], [7], [8], [9], [10], [11]. The dose-per-pulse (DPP) is in the range of 0.6 to 10 Gy that are orders of magnitude higher than in conventional radiotherapy. Typical pulse durations are in the range of 0.5 to 10 µs, resulting in extremely high instantaneous dose rates within one pulse (pulse dose rate) in the order of MGy/s. A recent review of the various electron beam parameters used to demonstrate the FLASH effect can be found in [12]. In order to exactly determine the parameters under which the FLASH effect occurs, an accurate dosimetry is essential. So far, mainly passive dosimeters like alanine dosimeters, radiochromic films, or thermo-luminescent dosimeter (TLD) are being used under these conditions [7], [13], [14], [15]. This is basically due to the fact that vented ionization chambers (IC) usually used for reference dosimetry in conventional radiotherapy show very large recombination losses under pulsed UHDR conditions [15], [16], [17], [18]. However, the use of passive dosimeters is complex, very time-consuming and usually lacking the traceability to primary standards. Thus, there is a need for active real-time dosimeters and traceability. With this, it is desirable to use ICs also under ultra-high DPP conditions as a secondary standard. The direct way to minimize recombination losses for a given IC geometry is to increase the electric field strength. Alternatively, decreasing general recombination effects can be achieved combining electrode distance reduction and chamber voltage increase [16]. Reducing the electrode distance has been shown to be more effective than increasing the chamber voltage. Based on theoretical considerations and experiments, it has been shown that a reduction of the electrode distance down to 0.25 mm can be well suited [19]. In this work, well-guarded vented parallel-plate ionization chambers (PPIC) with three different electrode distances are investigated experimentally and by means of simulations under ultra-high DPP conditions. The well-guarded design ensures that the electric field inside the sensitive volume is homogeneous. This avoids regions with lower field strength and therefore higher recombination losses as observed in [16]. Furthermore, this homogeneous electric field agrees to the model used for the numerical simulations. Since it has been shown that the standard theories based on Boags’s model of recombination fail at ultra-high DPP [16], [17], [18], the numerical approach of Gotz et al. [20] was used. Detailed simulation of the different effects contributing to the charge collection efficiency (CCE) have been performed. Furthermore, the numerical approach has been extended to account for charge multiplication.

## Methods

### Investigated parallel-plate ionization chambers

**Fig. 1:**
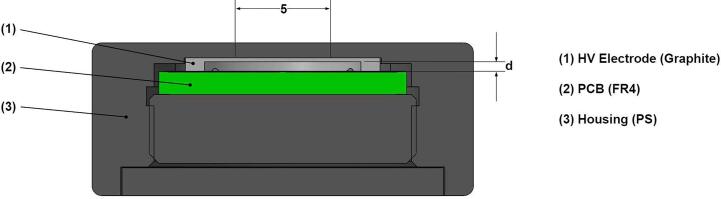
Cross section of the parallel-plate IC design with electrode distances d.

Prototypes of PPICs with different nominal electrode distances *d* of 1, 0.5 and 0.25 mm were manufactured by PTW for this investigation. The diameter of the measuring electrode is 5 mm. Therefore, the prototype with *d* = 1 mm has the same dimensions of the sensitive volume as the Advanced Markus chamber type 34045, which was already very well investigated under ultra-high DPP conditions [16], [21]. This serves as a benchmark for our investigation. The collecting electrode and the surrounding guard ring are realized by a carbon print on a circuit board. The guard ring is 2 mm wide and therefore two times greater than the largest electrode distance. Thus, it can be assumed that a homogeneous electric field within the collecting air volume is given. The electrode distance is defined by the depth *d* of the cavity in the high voltage electrode (see Fig. 1). This part is made of pure graphite, can be manufactured with high machine precision and is the only part that contributes to the tolerance of the electrode distance in this IC design. The housing is made of polystyrene (PS) and waterproof.

All used ICs were calibrated to absorbed dose-to-water with a Co-60 source under reference calibration conditions at the PTW secondary standard laboratory. The calibration coefficients *N_Co-60, Dw_* and the electrode distance of the different ICs are listed in Table 1. The nominal electrode distance *d_nominal_* was verified using micro-CT images. The measured distance between the electrodes *d_meas_* was determined and used in the following simulations.

**Table 1:** The electrode distances d, the calibration coefficients N_Co-60, Dw_, and the correction factors k_cross_ of the PPICs used in this investigation.

**IC ID**	**d_nominal_ (mm)**	**d_meas_ (mm)**	***N_Co-60, Dw_* (C/Gy)**	***k_cross_***
PPIC_1A	1	0.979	1.324E + 09	0.952
PPIC_1B	1	0.986	1.358E + 09	0.939
PPIC_0.5A	0.5	0.508	2.697E + 09	0.967
PPIC_0.5B	0.5	0.506	2.695E + 09	0.966
PPIC_0.25A	0.25	0.256	5.571E + 09	0.989
PPIC_0.25B	0.25	0.259	5.630E + 09	0.995

The dose measured with the IC $D_{\mathrm{IC}}$ was calculated using the following equation.

(1) $$D_{\mathrm{IC}}=\left(M-M_{0}\right)*N_{Co60,\;Dw}*k_{cross}*k_{T,P}*k_{P}$$

Here *M* is the reading of the dosemeter, *M_0_* is the reading of the dosemeter without irradiation. To account the deviation of the measurement condition from the calibration condition, especially with regard to the radiation quality (20 MeV electrons vs Co-60), the correction factors *k*_cross_ were applied. These factors were determined by a cross-calibration performed against calibrated alanine dosimeters using the 20 MeV electron beam at the lowest DPP used in this investigation. The well-established alanine dosimetry system at PTB, that has been standardized and extensively tested [22], [23], was used as the dose rate independent reference. The alanine dosimeter has been calibrated with Co-60. To account the used electron beam quality a correction factor of 1.014 was applied [23], [24].

Currently, correction factors *k*_Q_ to account for the beam quality dependence of these new prototypes under measurement conditions are not yet available. The air density is considered by the correction factor *k*_T,P_ and the polarity effect by the correction factor *k*_P_.

For a known reference dose value $D_{w}$, the CCE of the ionization chamber is given by the ratio of $D_{\mathrm{IC}}$ and $D_{w}$, which is the reciprocal of the correction factor *k*_S_ for incomplete charge collection due to ion recombination.

(2) $$\mathrm{CCE}=\frac{\left(M-M_{0}\right)*N_{Co60,\;Dw}*k_{cross}*{k_{T,P}*k}_{P}}{D_{w}}=\frac{1}{k_{S}}$$

### Measurement setup

The PTB's ultra-high pulse dose rate reference electron beam [25] was used for the measurements in this investigation. The accelerator including the beamline and the monitor system was described in detail by [26]. A 20 MeV electron beam with a pulse repetition frequency of 5 Hz and a pulse duration of 2.5 µs was used. Measurements were performed in a 30 ⨯ 30 ⨯ 30 cm^3^ water phantom with 1 cm thick walls made of PMMA and an entrance window of 0.8 mm polycarbonate. The surface of the phantom was positioned at 70 cm from the exit window of the horizontal beamline. This setup was already used in a previous study and is described in [25], [27]. The IC under test was placed at the reference depth $z_{\mathrm{ref}}$ according to TRS-398 [28]. The ionization current was measured using a Keithley 616 electrometer in current mode. To avoid high voltage peaks at the electrometer input an additional capacitor with 33 nF was inserted between detector and electrometer. Chamber voltages of 125 V, 250 V and 500 V were applied with both polarities. For each individual data point, 100 pulses were averaged. Thus, the statistical fluctuation uncertainties are less than 0.1 % and always within the symbol size of the figures presented in the results section. To determine the reference DPP, the beam current monitor was calibrated against alanine. The relative uncertainty of the alanine measurement is estimated to be 0.6 % with the largest contribution (0.5 %) attributed to the beam quality correction from ^60^Co radiation to electron beams [24]. The DPP is changed by varying the width of a slit aperture. Thus, a variation of the charge per electron beam pulse from 30 nC to 180 nC could be achieved. This corresponds approximately to a DPP range from 1 Gy to 5.4 Gy at $z_{\mathrm{ref}}$.

### Numerical approach

A numerical approach by solving a system of partial differential equations (PDE), taking into account charge creations by the radiation, their transport and reaction in an applied electric field was used to describe the theoretical behavior. A more detailed description and validation of this approach can be found in [16], [20]. Besides the determination of CCE, this method also allows the separation of the different effects like the fraction of ions and electrons, the distortion of the electric field and the ion recombination rates in the IC with temporal resolution based on the following equation system.

$\partial_{t}\rho_{+}=R-\frac{\alpha }{e}\rho_{+}\rho_{-}-div\left(\mu_{+}E\rho_{+}\right)-\frac{\beta }{e}\rho_{+}\rho_{e}$+$\alpha_{n}\rho_{e}$

$$\partial_{t}\rho_{-}=\gamma \rho_{e}-\frac{\alpha }{e}\rho_{+}\rho_{-}+div\left(\mu_{-}E\rho_{-}\right)$$

$\partial_{t}\rho_{e}=R-\gamma \rho_{e}+div{(\mu }_{e}E\rho_{e})-\frac{\beta }{e}\rho_{+}\rho_{e}$+$\alpha_{n}\rho_{e}$

$$E=-grad\left(\varphi \right)$$

(3) $$\rho_{+}-\rho_{-}-\rho_{e}=\varepsilon div\left(E\right)$$

The symbols in the above equations are defined as follow:

• $\rho_{+}$, $\rho_{-}$,$\rho_{e}$ are the unsigned densities of positive ions, negative ions and electrons
• R is the charge liberation rate due to irradiation
• α is the ion-ion recombination rate, β is the electron–ion recombination rate, γ is the electron attachment rate and e is the elementary charge
• $\mu_{+,}\mu_{-}{,\mu }_{e}$ are the mobilities of positive ions, negative ions and electrons
• φ is the electric potential and E is the electric field
• $\alpha_{n}$ is the first Townsend coefficient
• ɛ is the electric permittivity

All values for the above parameters were taken unchanged from Gotz et al. [20], [29]. The readers are referred to the original work for more details and a discussion of the associated uncertainties. To account for charge multiplication at high electric field strength the first Townsend coefficient $\alpha_{n}$ was added to the numerical equations. Due to the lack of experimental data in the range of electric field strength present in the PPICs under investigation, the coefficient for 50 % humid air and standard conditions for air pressure and temperature (1013 hPa and 20 °C) were determined using the software PyBoltz [30]. The PDE was solved by the method of lines. The spatial discretization is analogous to [20]. The resulting ordinary differential equation was solved using the package DifferentialEquations.jl [31] in the programming language Julia. To simulate measured currents, two approaches were implemented. The first approach simply counts the number of particles reaching the electrodes. While this approach gives the correct accumulated charge after sufficient time, it does not model temporal progress accurately. The reason is, that charge carriers already induce current, before they reach the electrodes. The Shockley-Ramo theory describes this phenomenon and was implemented in a second approach. In the case of a PPIC with electrode distance *d*, the current *I* induced by a particle moving with velocity *v* and charge *q* is given by:

(4) I=qv/d

In this work, all figures are based on the second approach, which takes this formula (Eq. (4)) into account.

## Results

### Charge collection efficiency

**Fig. 2:**
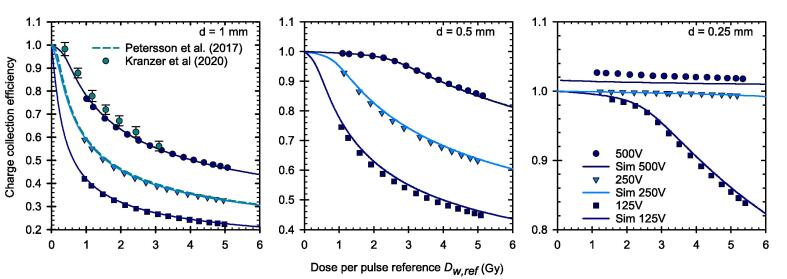
Charge collection efficiency of the PPICs with different electrode distances and three different chamber voltages. From left to right d = 1 mm, 0.5 mm, 0.25 mm. Experimental and simulated data as well as comparative data from the literature are presented. It should be noted that the scaling of the y-axis is adjusted from left to right.

The CCE could be experimentally determined by comparison of the dose reading against the reference dose. Also, at ultra-high DPP the CCE increases with decreasing electrode distance and increasing chamber voltage. This is shown for the PPICs with the three different electrode distances in Fig. 2. There is a good agreement between the simulated and the experimental data. Previous experimental data from the Advanced Markus IC type T34045 for *U* = 500 V and a logistic fit function [16], [17] for *U* = 250 V are also shown for the PPIC with 1 mm electrode distance for comparison. For the PPIC with the smallest electrode distance of 0.25 mm and a voltage of 250 V, the CCE is close to unity over the whole investigated DPP range exactly as predicted by the simulation. At 500 V, the CCE is approximately 2 % greater than unity which might be an indication for charge multiplication due to too high electric field strength. This is particularly pronounced for the lowest DPP and then decreases slightly with increasing DPP. The charge multiplication can be confirmed by the simulation, but the values are 1 % lower than the experimental values. The decrease with increasing DPP is also less pronounced in the simulation results.

**Fig. 3:**
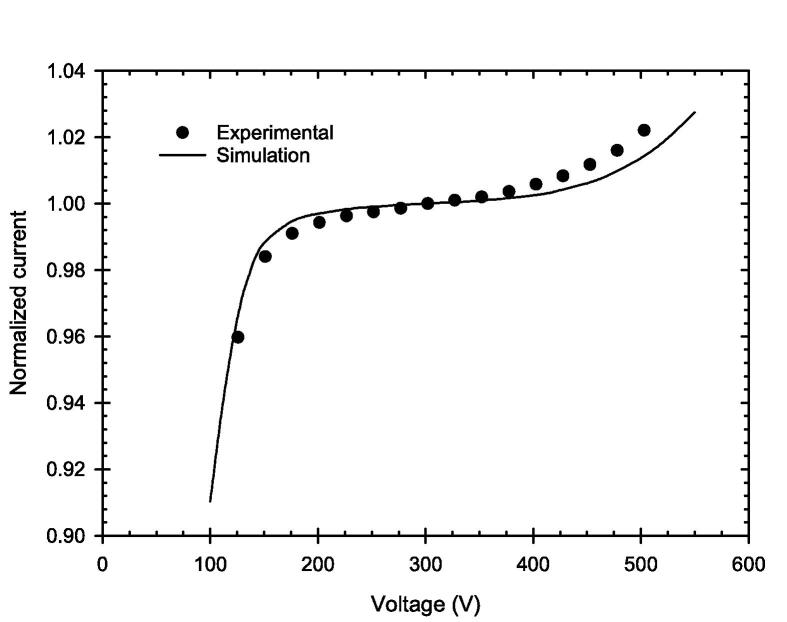
Experimental and simulated current–voltage characteristic of a prototype PPIC with 0.25 mm electrode distance (PPIC_0.25A) normalized to the current at 300 V at constant DPP of 2.78 Gy.

The charge multiplication can also be observed in the current–voltage characteristic at constant DPP for the prototype PPIC with 0.25 mm electrode distance in Fig. 3. The signal starts to increase clearly after 375 V from that within the plateau, considered as ionization chamber region in the current–voltage characteristic (normalized at 300 V), reaching the experimentally determined value of 1.02 at 500 V. Here, too, an underestimation of the charge multiplication can be seen in the simulation compared to the experimental data.

**Fig. 4:**
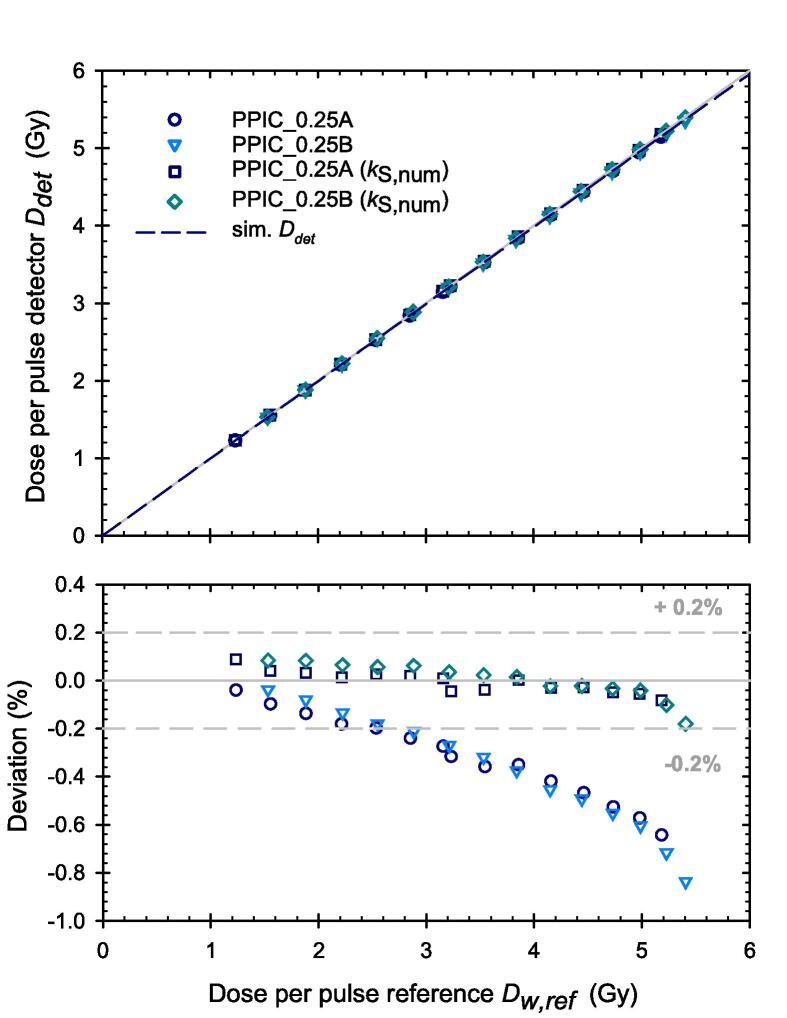
Measured DPP vs reference DPP for the PPICs with d = 0.25 mm at a voltage of 250 V with and without consideration of the correction factor k_S,num_. The deviation from linearity is shown in the lower panel.

From the results in Fig. 2, the smallest deviations of the measured dose calculated according to Eq. (1) from the reference dose were found for the PPICs with 0.25 mm electrode distance operated at 250 V. The measured DPP were plotted as function of the reference DPP in the upper panel of Fig. 4. There is only a slight deviation from linearity (up to 1 %) at the highest investigated DPP of 5.4 Gy (Fig. 4 lower panel). By applying the numerically determined ion recombination correction factor *k*_S,num_ given by the reciprocal of the simulated CCE, the deviations can be reduced to smaller than 0.2 % (Fig. 4, lower panel).

**Fig. 5:**
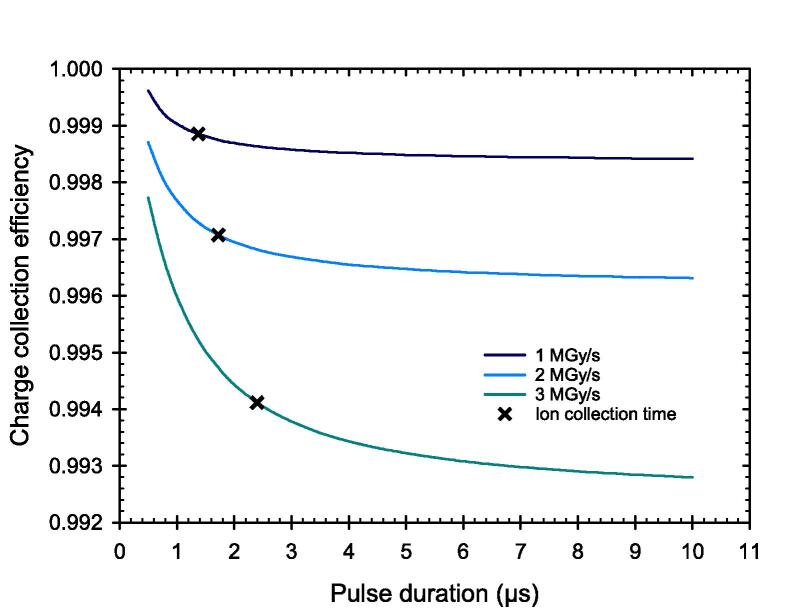
Simulated charge collection efficiency of the PPIC electrode distance 0.25 mm and chamber voltage 300 V versus the pulse duration for three different pulse dose rates. The time required for the slowest ions to pass the distance between the electrodes (ion collection time) is given for the respective pulse dose rates.

By means of the simulations, it has been shown that above a certain pulse length, an almost static state of the electric field occurs. This is accompanied by a constant CCE. The static state is approximately reached after a time which the slowest charge carriers need to pass the distance between the electrodes. This ion collection time was determined with the lowest ion velocity within the static state based on the simulations. Using the example of the PPIC with electrode distance 0.25 mm and chamber voltage 300 V, it can be seen, that the CCE decreases by less than 0.15 % from this point up to a pulse dose rate of 3 MGy/s and thus remains almost constant (Fig. 5).

### The role of electrode distance

**Fig. 6:**
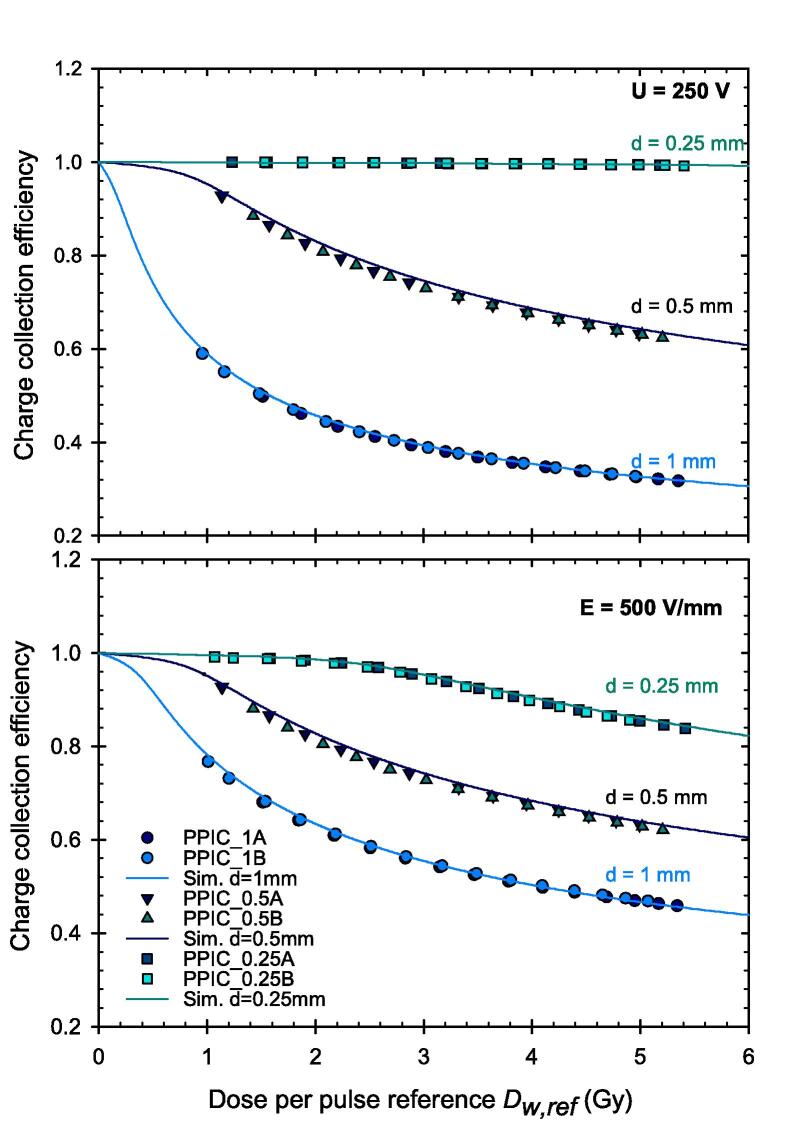
Charge collection efficiency of the different PPICs with the same chamber voltage U in the upper panel and the same electric field strength E in the lower panel.

The influence of the electrode distance can be elucidated by studying the CCE while maintaining the same chamber voltage. As shown by the results in the upper panel of Fig. 6 obtained using the same voltage of 250 V, decreasing the electrode distance leads to higher electric field strength and therefore higher CCE. There is a good agreement between the two samples A and B for each electrode distance. In the lower panel of Fig. 6, the voltage was chosen such that the electric field strength was the same (500 V/mm) for all ICs. Despite the same field strength, the PPIC with the smallest electrode distance shows the highest CCE indicating another mechanism, besides the field strength, through which the electrode distance influences the CCE.

Close examination on the results obtained using different PPICs with different electrode distances *d* and applied chamber voltages *U* reveals an invariance:

$$CCE\;\left(d_{1},\;U_{1}\right)\;\approx \;CCE\;\left(d_{2},\;U_{2}\right).$$

$if\;\frac{{d_{1}}^{2}}{U_{1}}=\frac{{d_{2}}^{2}}{U_{2}}.$

**Fig. 7:**
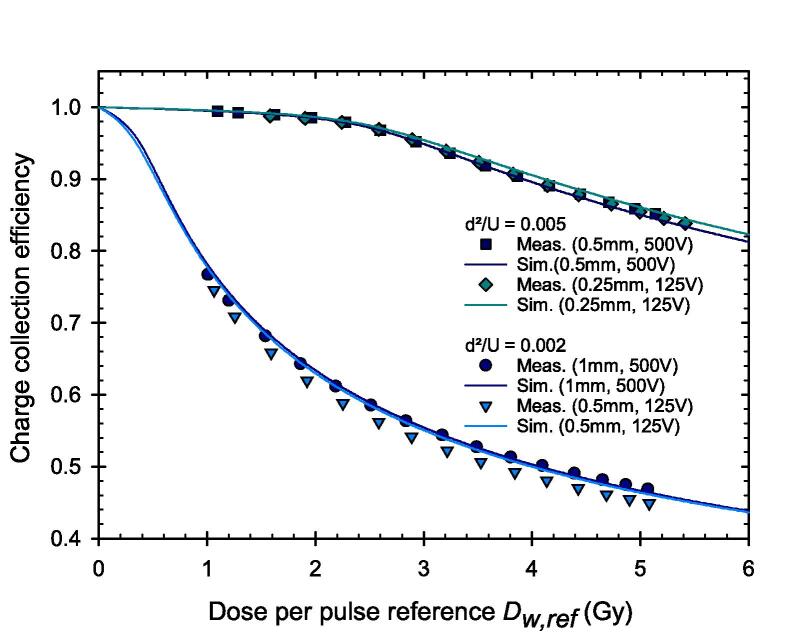
The CCE of the PPIC is invariant if the ratio of the square of the electrode distance and the applied voltage (d^2^/U) is equal.

In other words, two PPICs with electrode distances *d*_1_ and *d*_2_ operated at chamber voltages *U*_1_ and *U*_2_ irradiated under the same conditions with a pulsed beam without pulse overlapping will exhibit the same CCE if the ratios of the square of the electrode distance and the applied voltage of both chambers are equal. The validity of this scaling rule under UHDR condition is demonstrated in Fig. 7. In the lower group of data consisting of one PPIC with 1 mm electrode distance operated at 500 V and one PPIC with 0.5 mm operated at 125 V, the ratios *d*^2^/*U* in both cases correspond to 0.002. In the upper group of data consisting of one PPIC with 0.5 mm electrode distance operated at 500 V and one PPIC with 0.25 mm operated at 125 V, the ratios *d*^2^/*U* in both cases correspond to 0.005. Generally, a higher value of *d*^2^/*U* leads to a more favorable saturation behavior.

### Underlying recombination mechanisms

To further understand the underlying mechanism determining the CCE of the PPIC, different parameters were calculated using the numerical approach for the three electrode distances and a field strength of 500 V/mm. A pulse duration of 2.5 µs as used in the experiments with a DPP of 5 Gy were implemented in the calculations.

**Fig. 8:**
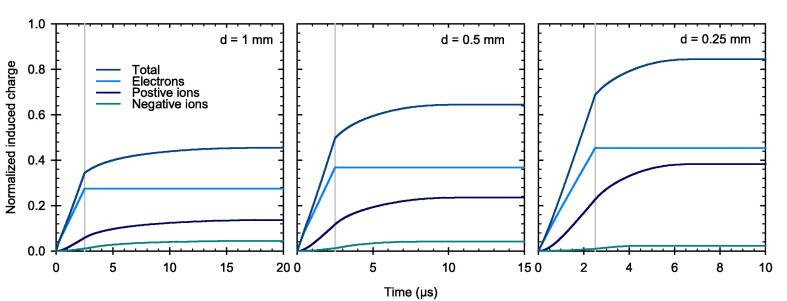
Induced charge over time, normalized to the total number of charge liberated by a pulse of 5 Gy and 2.5 µs duration. The end of the pulse is indicated by the grey line. PPICs with three different electrode distances d. Applied field strength 500 V/mm.

Fig. 8 shows the instantaneous induced charge of different species at the collecting electrode with respect to the time after the pulse started. The instantaneous induced charge is normalized to the total charge liberated by the pulse. At this ultra-high DPP, a large fraction of free electrons will contribute directly to the measured signal, where only a minor portion of the liberated electrons will form negative ions through attachment. Due to their high mobility, all the free electrons reach the electrode at the end of the pulse.

**Fig. 9:**
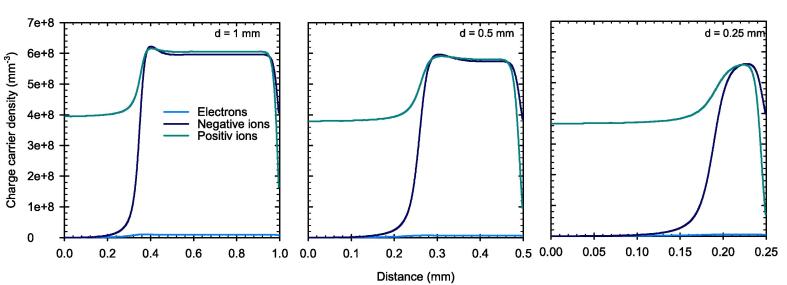
Charge carrier density as a function of distance from the negative electrode of the PPIC with three different distances d at the end of the pulse with a DPP of 5 Gy and 2.5 µs duration. The applied field strength corresponds to 500 V/mm.

The different collection time of the charge carriers create a temporal and spatial charge imbalance within the sensitive volume of the PPIC. The densities of the different charge carriers at the end of the pulse as a function of the distance from the negative electrode are plotted in Fig. 9 for three electrode distances.

**Fig. 10:**
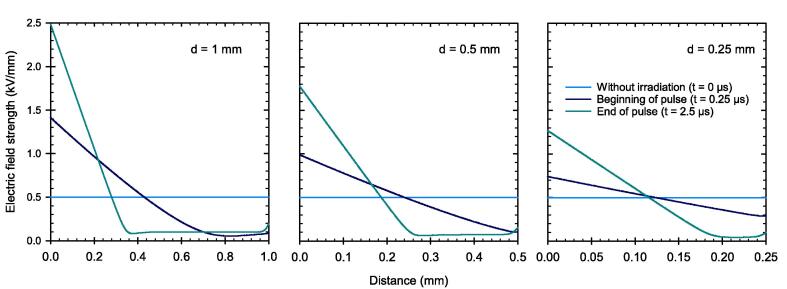
Electric field strength inside the PPIC with electrode distances d at different instances. Before the pulse (t = 0 µs), at the beginning of the pulse (t = 0.25 µs), and at the end of the pulse (t = 2.5 µs) with a DPP of 5 Gy.

The charge carrier imbalance causes disturbance in the local electric field. The longer the pulse with a duration of 2.5 µs lasts, the stronger is this distortion. Fig. 10 shows the electric field between the electrodes at three instances: before the pulse (t = 0 µs), at the beginning of the pulse (t = 0.25 µs), and at the end of the pulse (t = 2.5 µs). Shortly after the pulse begins, the electric field deviates from the initial constant field strength (500 V/mm). At the end of the pulse, the electric field collapses almost completely, that is, approaching zero, in part of the sensitive volume adjacent to the positive electrode. The results in Fig. 10 show that the effect is more pronounced for a larger electrode distance. For the electrode distance of 1 mm, the region with vanishing electric field cover almost 65 % of the total sensitive volume (left panel).

**Fig. 11:**
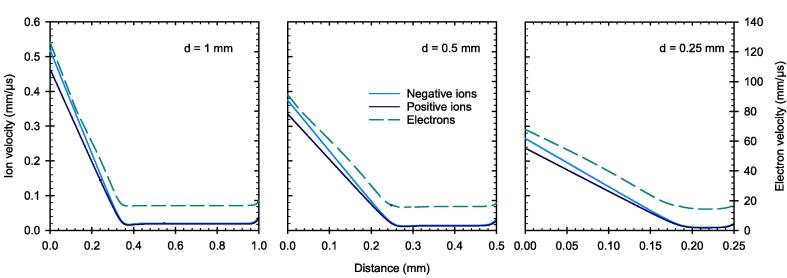
Charge carrier velocity between the electrodes of the PPIC with electrode distances d at the end of the pulse with a DPP of 5 Gy and 2.5 µs duration. The applied field strength corresponds to 500 V/mm.

As a result of the field distortion, the velocity of the charge carriers is reduced the further away they are from the negative electrode as presented in Fig. 11 at the end of the 2.5 µs pulse. This is especially true for the charge carriers in the PPIC with 1 mm electrode distance, where the ions are brought almost to a complete standstill within a large part of the sensitive volume, travelling at a velocity of approximately 0.02 mm/µs (left panel). Again, it can be seen that the electrons move at significantly higher velocity than the ions.

**Fig. 12:**
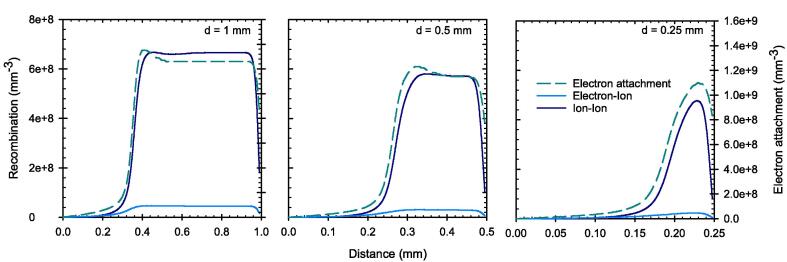
Recombination and attachment between the electrodes of the PPIC with electrode distances d at the end of the pulse with a DPP of 5 Gy and 2.5 µs duration. The applied field strength corresponds to 500 V/mm.

This reduction of the charge carrier velocity leads to an increased electron attachment and recombination rate, where the ion-ion recombination is the dominant effect here as shown in Fig. 12 at the end of the pulse. Consequently, despite the initially constant electric field, the CCE of the chamber is dependent on the electrode distance, where the lowest CCE is associated with PPIC with the largest electrode distance (1 mm in our case).

## Discussion

The CCE of PPICs with three different electrode distances have been determined experimentally. The CCE increases with decreasing electrode distance that confirms the results of a previous work [16]. Due to the well-guarded design of the PPICs, the electric field can be considered as homogeneous and thus fulfills the assumptions of the numerical approach. The agreement between experimental and simulated data is thus also given for small electrode distances in this investigation.

For the smallest PPIC with an electrode distance of 0.25 mm and an applied chamber voltage of 250 V, the CCE is shown to be greater than 99 % even for the highest DPP of 5.4 Gy investigated in this work. Increasing the chamber voltage within the ionization chamber region of the current–voltage curve could increase the CCE again. Based on the simulations, a CCE of 99.7 % can be achieved for the highest DPP of 5.4 Gy with a chamber voltage of 300 V, without getting charge multiplication. This is also true for the lowest DPP studied, where at 500 V the observed deviation (CCE greater than 1) was most pronounced (Fig. 2). This finding is in agreement with the simulations of Gómez et al. [19], which have shown CCE of 99 % up to a DPP of 7.5 Gy for a PPIC with d = 0.25 mm, U = 300 V and a pulse duration of 2.5 µs. The further increase, however, would lead to charge multiplication, as can be seen in Fig. 3 in both the measured and simulated current–voltage characteristic curves.

If the PPICs with the different electrode distances are operated with a chamber voltage that leads to the same electric field strength within in the PPIC, the ionization chambers still show a clearly different CCE. In this case, the PPIC with the smallest electrode distance provides the highest CCE. This shows that the electrode distance is the crucial parameter under ultra-high DPP conditions which enters quadratically. It was demonstrated that the simulated CCE for different combinations of electrode distance and chamber voltage agrees well with the experimental data. If the correction factor *k*_S,num_ is determined from the simulation as reciprocal of the CCE for the individual PPIC and the specific beam parameters and applied to determine the dose, agreement with the reference dose can be achieved with a maximum deviation of less than 0.2 % as shown for the PPICs with d = 0.25 mm and U = 250 V.

Based on the simulations, it has been shown that the high fraction of free electrons has a decisive influence on the ionization chamber’s behavior under ultra-high DPP conditions. Due to their high velocity, the free electrons reach the collecting electrode much faster than the ions, resulting in a temporal and spatial charge carrier imbalance within the collecting volume. As a result, the electric field is distorted in a way that the field strength decreases towards the positive electrode. In this low field region, the velocity of the ions is considerable reduced causing an increased recombination rate. The effect is shown to be more prominent for larger electrode distance. For pulse durations greater than the 2.5 µs as used in this study, a steady state of the electric field distortion will occur after a time on the order of the collection time of the slowest charge carriers. When this state is reached, the CCE will remain constant. The PPIC with 0.25 mm and 300 V chamber voltage, is very close to this condition, so that a longer pulse duration at constant dose rate during the pulse, will almost not cause any degradation of the CCE. This was shown by the simulations up to a pulse dose rate of 3 MGy/s. The observation is important to assess the validity of published correction factors for other pulse lengths and must be considered in the application.

The detailed numerical simulations have revealed the underlying mechanisms leading to the experimental observed behavior of the PPICs with different electrode distances.

In a recently published paper, Di Martino et al. [32], describe the theory and conceptual design of a gas-filled ionization chamber. By using the noble gas argon at extremely low pressures, the disturbance of the electric field due to the high charge carrier density in a vented ionization chamber as described above is avoided. This theoretically allows a linear behavior up to a DPP of 40 Gy at a pulse length of 4 µs within an assumed relative uncertainty of 4 %. As described in their paper, such a chamber design has extremely high engineering requirements. In particular, if the chamber is also to be used to determine the absorbed dose to water in a water phantom. In addition, there is the question of calibration and traceability to a primary standard.

## Conclusions

Vented PPICs with very small electrode distances are a promising tool for real-time dosimetry in FLASH radiotherapy. It was shown that with an electrode distance of 0.25 mm and a well-chosen chamber voltage, a CCE greater than 99 % can be achieved up to a DPP of 5.4 Gy at a pulse length of 2.5 µs. Thus, it is possible to correct the remaining recombination losses using the correction factors *k*_S,num_ derived from the numerical simulation. Our results support the application of ionization chambers according to the common codes of practice like TRS-398, TG-51 or DIN 6800–2 under UHDR with pulsed electron beams that would allow traceability to primary standards.

## Declaration of Competing Interest

Rafael Kranzer and Jan Weidner are PTW employees.
